# Selective biological activity of parthenolide derivatives — stizolin, stizolicin, and izospiciformin — isolated from the leaves of *Stizolophus balsamita*, in different breast cancer molecular subtypes *in vitro*

**DOI:** 10.3389/fphar.2026.1799963

**Published:** 2026-05-11

**Authors:** Joanna Nawrot, Ewa Totoń, Iga Dziechciowska, Agnieszka Boruta, Małgorzata Gołębiowska, Małgorzata Idzik, Mariusz Kaczmarek, Błażej Rubiś, Justyna Gornowicz-Porowska, Natalia Lisiak

**Affiliations:** 1 Department and Division of Practical Cosmetology and Skin Diseases Prophylaxis, Poznan University of Medical Sciences, Poznan, Poland; 2 Department of Clinical Chemistry and Molecular Diagnostics, Poznan University of Medical Sciences, Poznan, Poland; 3 Poznan University of Medical Sciences - Students, Poznan, Poland; 4 Department of Cancer Diagnostics and Immunology, Gene Therapy Unit, Greater Poland Cancer Centre, Poznan, Poland

**Keywords:** apoptosis, autophagy, breast cancer, cell viability, parthenolide derivatives

## Abstract

**Introduction:**

Breast cancer is the most frequently diagnosed malignant tumor and one of the leading causes of cancer deaths. Combination therapies, chemotherapy, and hormone therapy have indeed revolutionized the treatment of breast cancer, but they have not eliminated the occurrence of side effects. Among different anticancer strategies, plant-derived compounds appear to play a critical role in both prevention and therapy. They have been utilized in folk medicine for years, revealing anti-inflammatory, antimigraine, and anticancer properties. In particular, parthenolide derivatives have been shown to act as adjuvant agents in the treatment of various malignancies. Especially when provided with modifications that increase their bioavailability and stability.

**Methods:**

Here, we present the cell type-selective biological activity of parthenolide derivatives—stizolin, stizolicin, and izospiciformin—isolated from the leaves of *Stizolophus balsamita*, in a panel of three different molecular subtypes of breast cancer cell (MCF7, MDA-MB-231, and SK-BR-3). Viability, clonogenic potential, apoptosis, and autophagy processes have been verified to explain the biological activity of the studied natural compounds.

**Results:**

The highest biological activity was demonstrated by the stizolin (with the IC50 from 1.3 to 4.3 μg/mL depending on cell line), which exhibited antiproliferative, proapoptotic, and proautophagic properties in the studied breast cancer cells, particularly in the HER2-positive breast cancer cell line (SK-BR-3), demonstrated by PARP1/2 cleavage, Bax/Bcl2 status increase, and confirmed by LC3II/LC3I, mTOR, and p62 proteins alterations.

**Conclusion:**

Our results indicate that the studied compounds exhibit compound-dependent biological activity, and selectivity across different molecular subtypes of breast cancer.

## 1 Introduction

Breast cancer poses a serious health problem for women around the world. It is the most frequently diagnosed malignant tumor and one of the leading causes of cancer deaths. Data published in Nature Medicine shows that 1 in 20 women worldwide will be diagnosed with breast cancer in their lifetime, and that if current trends continue, by 2050, there will be 3.2 million new breast cancer cases and 1.1 million breast cancer-related deaths per year (Kim et al., 2025).

The risk factors of breast cancer development comprise female gender (Valentini et al., 2024), mature age (Obeagu and Obeagu, 2024), a family history of breast cancer (especially presence of ovarian cancer–and especially those caused by hereditary *BRCA1* and *BRCA2* mutations) (Casaubon et al., 2023), but also mutations in highly penetrant breast cancer genes, like *CDH1*, *PTEN*, *STK11*, and *TP53* (Pal et al., 2024). Moreover, the race (Ribeiro et al., 2025), exposure to endogenous hormones (particularly estrogen and progesterone), and early age at menarche (García-Sancha et al., 2025) are other significant risk factors of breast cancer development. Modifiable risk factors include smoking, physical activity (Cohen et al., 2023), and higher BMI (body mass index), which is associated with more aggressive features of the tumor (Tzenios et al., 2024).

The hormonal and receptor status of breast cancer was used to divide this disease into the four main molecular subtypes: i) estrogen receptor-positive - luminal A and ii) luminal B, iii) HER2+, and iv) triple-negative/basal breast cancers (Lakhani et al., 2012). These features, together with the degree of advancement and malignancy and the patient’s general condition, determine the therapeutic strategy (Xiong et al., 2025).

With the development of oncological diagnostics, new methods of breast cancer treatment are being introduced. Combination therapies, chemotherapy, and hormone therapy have indeed revolutionized the treatment of breast cancer, but they have not eliminated the occurrence of side effects. Thus, there is a need to develop new, combined, and personalized strategies that are effective and safe for patients. A plant-based approach appears to be a critical trend in both prevention and therapy.

**FIGURE 1:**
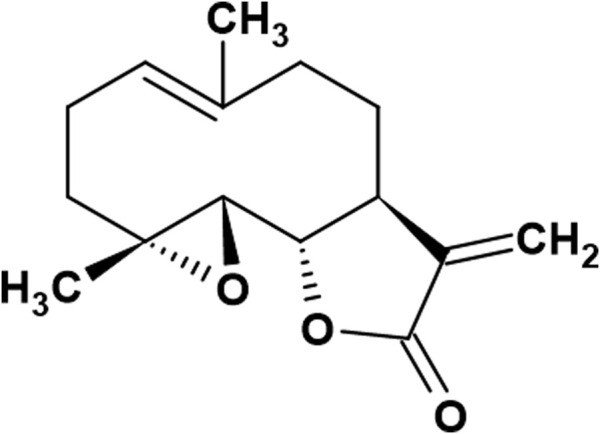
Chemical structure pf parthenolide (PN) (prepared according to Liu et al., 2024).

Plant-derived compounds have been utilized in folk medicine for years, and many scientists have drawn renewed attention to their potential use in anticancer therapy. These include sesquiterpenes with diverse structures and broad biological activities. Over the years, numerous properties of these compounds, including anti-inflammatory, antifungal, bactericidal, and anticancer properties, have been revealed. One such naturally occurring compound is parthenolide (PN) (Figure 1; Liu et al., 2024). This sesquiterpene lactone with a chemical formula of C15H20O3, is commonly found in many plants, such as *Tanacetum parthenium* or *Tanacetum vulgare* (Michalak et al., 2024). It was initially isolated from plants in the *Asteraceae* family in the 1970s. The structure of parthenolide contains an α-methylene-γ-lactone ring and an epoxy group, which can interact with the nucleophilic sites of biological molecules, affecting cellular signaling pathways, including the induction of oxidative stress and apoptosis, Focal Adhesion Kinase 1 (FAK1) signaling, and Hypoxia-Inducible Factor 1α (HIF-1α) signaling (Zarei et al., 2025; Berdan et al., 2019).

Recent studies have demonstrated that parthenolide exhibits anti-inflammatory properties and also plays a role in cancer response to therapy (Li et al., 2025).

It attenuates the pathogenicity of cancer and increases the sensitivity of various types of cancer to chemotherapy or radiation. It was reported to show cytotoxic potential in liver, colorectal, thyroid, pancreatic, myeloma, prostate, and breast cancer cells, and to be very well-tolerated by humans (Denda et al., 2024). Importantly, studies indicate that parthenolide can induce apoptosis in myeloid leukemia cells (AML) without affecting normal hematopoietic cells (Parthenolide was also used in preclinical studies to treat relapsed leukemia associated with multidrug resistance, which is driven by leukemia stem cells (LSCs) (Zarei et al., 2025). The limitations of parthenolide use are repeatedly emphasized due to its low bioavailability and stability (Liu et al., 2023). Thus, much attention is paid to the anticancer potential of not only parthenolide but also its derivatives, such as dimethylaminoparthenolide (DMAPT), which have been the subject of preclinical *in vitro* and *in vivo* studies (Zarei et al., 2025). This parthenolide derivative demonstrated radiosensitizing properties in prostate cancer cells (PC-3 and DU145) and breast cancer stem-like cells, while also exhibiting radioprotective activity in normal cells (Uzun et al., 2025).

However, further studies regarding the anticancer potential of parthenolide have identified alternative mechanisms of action, which we also demonstrate in our studies. These activities lead to cancer cell death by modulating breast cancer cell proliferation, apoptosis, and autophagy.

## 2 Materials and methods

### 2.1 Plant material

**FIGURE 2:**
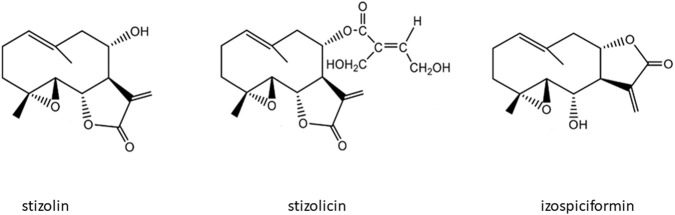
Structure of parthenolide derivatives: stizolin, stizolicin, and izospiciformin.

The methanolic extract under study was prepared from the aerial parts (leaves) of *Stizolophus balsamita* (Lam.) K. Koch (*Asteraceae*) in the Department of Medicinal and Cosmetic Natural Products, University of Medical Sciences in Poznan (Poland), and tested compounds (stizolin, stizolicin, izospicifromin) (Figure 2) were isolated there from the CH2CL2 extract.

The complete extraction procedure, isolation method, and detailed structural identification of compounds have been previously described (Nawrot et al., 2019a).

Seeds of *S. balsamita* were provided by the Botanical Garden (Tehran, Iran) and aerial parts (grown from the seeds in the Garden of Medicinal Plants at the Department of Medicinal and Cosmetic Natural Products, University of Medical Sciences in Poznan, Poland, where the voucher specimens (No 42/2014) is deposited) were collected in the flowering period (September) and dried at room temperature. The extract studied in our paper is a mixture of seven sesquiterpene lactones: balsamin, stizolin, stizolicin, 9α-hydroxyparthenolide, izospiciformin, 8α-E-(4′-hydroxy)-sene-cioyloxy-9α-hydroxyparthenolide, and 11βH,13-dihydrostizolicin (Nawrot et al., 2019b).

### 2.2 Reagents

DMSO (Sigma-Aldrich, St Louis, MO, United States), SDS (Sigma-Aldrich, St Louis, MO, United States), MTT (Sigma-Aldrich, St Louis, MO, United States), trypsin-EDTA (Sigma-Aldrich, St Louis, MO, United States), FBS (Corning, NY, United States), RPMI 1640 medium (Corning, NY, United States), McCoy’s 5a medium (Corning, NY, United States), Ham’s/DMEM medium (Corning. NY, United States), horse serum (ATCC, Manassas, VA, United States) bovine insulin (Sigma-Aldrich, St Louis, MO, United States), hydrocortisone (Sigma-Aldrich, St Louis, MO, United States), hEGF (Sigma-Aldrich, St Louis, MO, United States), RIPA lysis buffer (Thermo Fisher Scientific, Waltham, MA, United States), protein cocktail inhibitors (Thermo Fisher Scientific, St Louis, MO, United States), propidium iodide (Sigma-Aldrich, St Louis, MO, United States), ribonuclease A (Sigma-Aldrich, St Louis, MO, United States), PBS (BioShop, Rzasnik, Poland), antibodies: anti-PARP1/2 (Cell Signaling Technology, Danvers, MA, United States), anti-Bax (Cell Signaling Technology, Danvers, MA, United States), anti-Bcl2 (Cell Signaling Technology, Danvers, MA, United States), anti-MAPLC3 (Cell Signaling Technology, Danvers, MA, United States), anti-p62/SQSTM (Cell Signaling Technology, Danvers, MA, United States), anti-mTOR (Cell Signaling Technology, Danvers, MA, United States), anti-GAPDH (Santa Cruz Biotechnology, Dallas, TX, United States), rapamycin (Sigma-Aldrich, St Louis, MO, United States), doxorubicin (Sigma-Aldrich, St Louis, MO, United States), gefitinib (Cell Signaling Technology, Danvers, MA, United States), monodansylcadaverine (Sigma-Aldrich, St Lou-is, MO, United States), anti-fatty acid powder milk (Sigma-Aldrich, St Louis, MO, United States), bovine serum albumin (BioShop, Rzasnik, Poland), TRIS (BioShop, Rzasnik, Poland), Tween 20 (Sigma-Aldrich, St Louis, MO, United States), sodium persulfate (BioShop, Rzasnik, Poland), TEMED (BioShop, Rzasnik, Poland), acrylamide-bisacrylamide (Sigma-Aldrich, St Louis, MO, United States), Bradford reagent (Sigma-Aldrich, St Louis, MO, United States).

### 2.3 Cell lines and cell cultures

The human breast cancer cell lines: MCF7 (ER+, PR+, HER2-), MDA-MB-231 (ER-, PR-, HER2-), SK-BR-3 (ER-, PR-, HER2+), and MCF-12A human non-tumorigenic breast cell line (ER+, PR+, HER2-) were obtained from the American Type Culture Collection (ATCC, HTB-22, HTB-26, HTB-30, CRL-10782, respectively). The MCF7 and MDA-MB-231 cells were maintained in RPMI 1640 medium supplemented with 10% fetal bovine serum. The SK-BR-3 cell line was cultured in McCoy’s 5a medium supplemented with 10% fetal bovine serum. The MCF-12A cells were maintained in Ham’s/DMEM medium supplemented with 5% horse serum, bovine insulin (500 ng/mL), hydrocortisone (1 μg/mL), and hEGF (20 ng/mL). The cells were cultured in 5% CO_2_ at 37 °C and 100% humidity. The cells were passaged with medium changes every 3–4 days.

### 2.4 Viability assay

A total of 5 × 10^3 growing breast cancer and nontumorigenic breast cells were seeded into each well of the 96-well plates, and compounds (stizolin, stizolicin, and izospiciformin) or *S. balsamita* leaves extract were added at concentrations ranging from 1 to 100 μg/mL for 24, 48, and 72 h. Two duplicates were prepared for each concentration, with a total volume of 100 µL per well. The solvent, DMSO at a concentration of 0.25% was also used as a control (Sigma-Aldrich, St. Louis, MO, United States). Next, 10 µL of MTT solution (5 mg/mL) was added to each well. The plates were incubated at 37 °C for 4 h, then 100 µL of solubilization buffer (10% SDS in 0.01 M HCl) was added, as previously described (Romaniuk-Drapała et al., 2025). Cell viability was quantified spectrophotometrically (at 570 nm, with a reference wavelength of 690 nm) using a Labsystems Multiscan RC (Thermo, Champaign, IL, USA). Each experiment was repeated three times in duplicates, IC50 values were calculated using CompuSyn software (ComboSyn, Inc., Paramus, NJ, United States), and the standard deviation was calculated using Microsoft Excel software (Microsoft, Redmond, WA, United States).

The selective activity of an active compound can be expressed as a selectivity index (SI), which is determined by comparing its cytotoxic activity in normal cells vs. cancer cells. Thus the SI was calculated as the ratio of the IC50 for MCF-12A cells vs. IC50 for the corresponding cancer cells (MCF7, MDA-MB-231, and SK-BR-3), using the following equation:

SI=IC50 for normal cell line /IC50 for cancer cell line

SI values above 1.00 indicate that the tested compound exhibited higher selectivity for cancer cells than for normal cells (Tronina et al., 2023). SI ≤1.00 indicates that the compound is nonselective. The selectivity indexes of the tested compounds are presented in Table 2.

### 2.5 Colony-forming assay

For the colony-forming assay (clonogenic), MCF7, MDA-MB-231, and SK-BR-3 cells were plated at a density of 200 cells/well in 6-well plates and allowed to adhere for 24 h. They were then treated with stizolin, stizolicin or izospiciformin at three concentrations corresponding to 0.5×, 1×, and 1.5× IC50 for 24 h (Table 3). After the specified time point, the media were replaced, and the cells were grown for an additional 10 days, with one media change on the fourth day. The colonies formed were fixed with 4% formaldehyde (37 °C for 15 min) and stained with crystal violet (0.5% (w/v) for 1 h at 25 °C), as previously described (Lisiak et al., 2023). The wells were then washed with distilled water, air-dried, and the colonies were counted. The results were graphically presented. The experiment was repeated three times.

### 2.6 Cell cycle analysis

Analysis by Flow Cytometry of cultures of MCF7, MDA-MB-231, and SK-BR-3 cells was performed either with or without treatment with the indicated concentrations of stizolin, stizolicin or izospiciformin for 24 h. As a positive control for cell cycle alterations, gefitinib at 20 μM/mL has been used. The cells were collected using 0.25% trypsin (Sigma-Aldrich, St. Louis, MO, United States), then washed and resuspended in 100 µL PBS containing 50 μg/mL propidium iodide and 25 µL of ribonuclease A (10 mg/mL; Sigma-Aldrich, St. Louis, MO, United States). Flow cytometry analysis was performed after 1 h of incubation, as described previously (Lisiak et al., 2014; FACScan, BectonDickinson, Franklin Lakes, NJ). The percentages of the cell population in the subphases G1, S, and G2 were calculated from the histograms. The experiment was repeated twice in duplicates.

### 2.7 Propidium iodide (PI) and monodansylcadaverine (MDC) staining–autophagy/apoptosis dual staining assay

The PI/MDC staining assay was performed according to the manufacturer’s protocol (Promega, Madison, WI, USA). Briefly, cells were subcultured in 96-well plates at a density of 1 × 10^5 cells per well and incubated in appropriate media with stizolin, stizolicin or izospiciformin for 24 h. As a positive apoptosis control, gefitinib (20 µM) was used, and as a positive autophagy control, rapamycin (50 nM) was applied. After treatment, the cells were washed twice with PBS, incubated with 0.05 mM monodansylcadaverine (MDC) (Sigma-Aldrich, St Louis, MO, USA) in PBS at 37 °C for 10 min, and then washed three times with PBS at room temperature (RT). Next, cells were incubated with a propidium iodide solution (50 μg/mL) for 5 min at room temperature, and then washed three times with PBS. The mean fluorescence intensities (MFIs) from intracellular MDC and PI were measured with the plate reader with the excitation wavelength of 335 nm and an emission wavelength of 512 nm for MDC, and with the excitation wavelength of 536 nm and an emission wavelength of 617 nm for PI, respectively (Enspire, Perkin Elmer, Waltham, MA, USA). The results are representative of three independent experiments ([MFIs] ± SD) and are presented as relative values compared with untreated control cells.

### 2.8 Immunodetection

The MCF7, MDA-MB-231, and SK-BR-3 cells were treated for 24 h with three concentrations of stizolin, stizolicin or isospiciformin, i.e., 0.5×IC50, 1×IC50, and 1.5×IC50 (Table 3). Whole cell extracts were prepared using a modified RIPA lysis buffer (50 mM Tris-HCl, pH 8.0, 150 mM NaCl, 1% NP40, 0.1% SDS, 100 mM PMSF, 25 μg/mL Na3VO4, 25 μg/mL NaF, protease cocktail inhibitors). The protein concentration was measured using a Bradford assay (Sigma-Aldrich, St Louis, MO, United States), and 40 µg of each extract was loaded onto SDS-PAGE gels. Western blotting was performed according to standard procedures using a PVDF membrane (Pierce Biotechnology, Rockford, IL, USA), as previously described (Romaniuk-Drapała et al., 2021). The following antibodies were used for detection: anti-PARP, anti-Bax, anti-Bcl2, anti-MAPLC3, anti-SQSTM/p62, and anti-mTOR (all from Cell Signaling, Boston, MA, USA), and anti-GAPDH (Santa Cruz Biotechnology, Dallas, TX, United States); 1 μg/mL of each primary antibody was used in the blotting solution. The proteins were visualized using iBright Imaging System (ThermoFisher Scientific, Waltham, MA, United States) and SuperSignal® West Pico Chemiluminescent Substrate (Pierce Biotechnology, Rockford, IL, United States). The optical density (Arbitrary Units) of the bands was measured using LabWorks software (UVP, Upland, CA, United States). Representatives of the two experiments are shown in Figures 7A–C, 9A–C.

### 2.9 Statistical analysis

The data shown are means from at least three separate experiments, unless otherwise specified. Statistical analysis was performed using one-way analysis of variance (ANOVA) together with Tukey׳s multiple comparison test (GraphPad Prism 5, San Diego, CA, United States). P < 0.05 was considered to be indicative of a significant difference.

## 3 Results

### 3.1 Effect of the *Stizolophus balsamita* extract and selected compounds on breast cells’ viability

**FIGURE 3:**
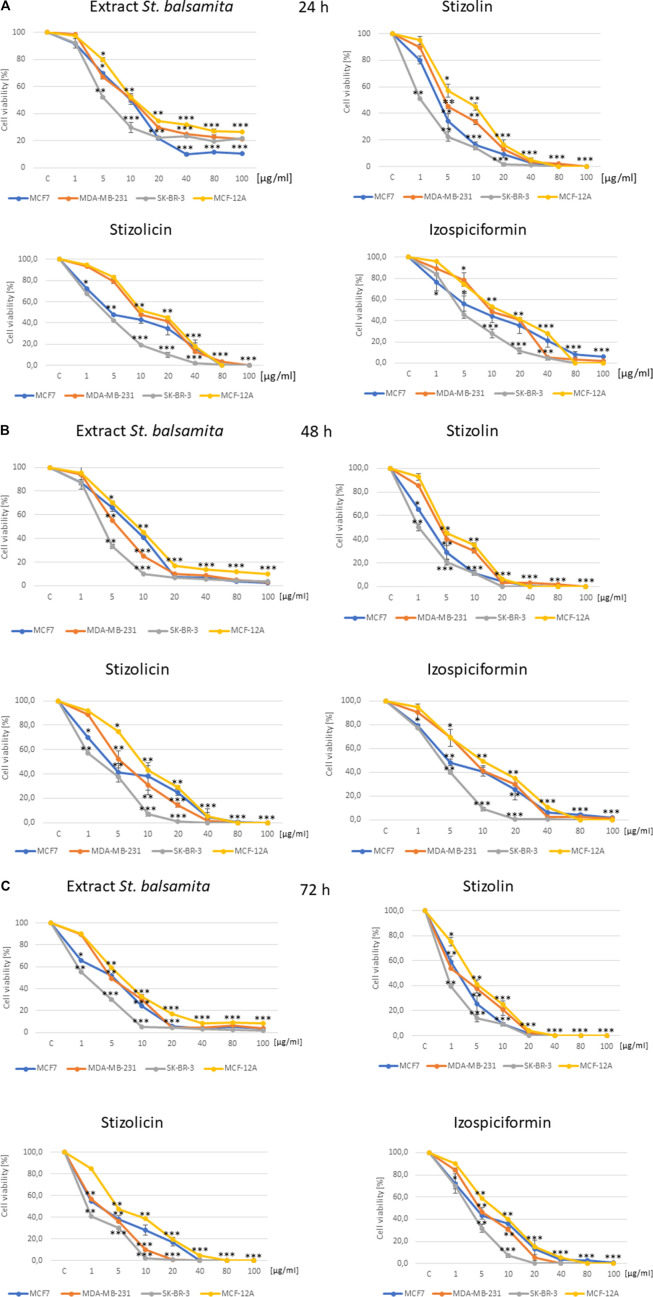
Assessment of Stizolophus balsamita extract, stizolin, stizolicin, and izospiciformin on breast cancer cells’ viability. MCF7, MDA-MB-231, SK-BR-3, and MCF-12A cells were treated for 24 **(A)**, 48 **(B)**, or 72 **(C)** h with a wide range of concentrations of studied compounds, i.e., 1–100 µg/mL. The mean ± SD from three experiments is shown. Statistically significant differences versus control samples are shown: *p < 0.05; p < 0.005; *p < 0.001.

Four breast cell lines were treated with different concentrations (1–100 μg/mL) of the *S. balsamita* extract, stizolin, stizolicin or izospiciformin for 24, 48, or 72 h. The extract evaluated in our study comprises seven sesquiterpene lactones: balsamin, stizolin, stizolicin, 9α-hydroxyparthenolide, izospiciformin, 8α-E-(4′-hydroxy)senecioyloxy-9α-hydroxyparthenolide, and 11βH,13-dihydrostizolicin. However, stizolin, stizolicin, and izospiciformin are dominant components of the extract; thus, they were chosen for further analysis (Nawrot et al., 2019b). Treatment of the studied cells with the extract revealed that the most sensitive cells were the HER-2-positive breast cancer cells, SK-BR-3, with an IC50 of 6.7 μg/mL after 24 h (Figure 3). The triple-negative breast cancer cells MDA-MB-231 was the least sensitive to the extract, with an IC50 of 12.8 μg/mL after 24 h of incubation (Table 1).

**TABLE 1:** The IC50 values ± S.D. of stizolin, stizolicin, izospiciformin, and Stizolophus balsamita extract in MCF7, MDA-MB-231, SK-BR-3, and MCF-12A cells. Cells (5 × 10^3/well) were treated for 24, 48, and 72 h. The mean of three experiments, each in duplicate, is shown. The results are shown relative to control (untreated) cells.

Cell line	Time [h]	IC_50_ ± S.D. [µg/mL]				
		Stizolin	Stizolicin	Izospiciformin	Extract	Doxorubicin
MCF7	24	2.7 ± 0.2	4.6 ± 0.15	9.3 ± 0.5	10.0 ± 0.8	14.9 ± 0.8
	48	2.1 ± 0.1	4.4 ± 0.2	4.7 ± 0.3	7.3 ± 0.5	0.25 ± 0.05
	72	2.0 ± 0.09	3.8 ± 0.2	3.8 ± 0.2	6.2 ± 0.3	0.06 ± 0.01
MDA-MB-231	24	4.3 ± 0.4	8.3 ± 0.4	8.5 ± 0.6	12.8 ± 0.6	7.0 ± 0.03
	48	3.7 ± 0.2	5.4 ± 0.3	6.9 ± 0.5	6.0 ± 0.2	0.24 ± 0.02
	72	1.4 ± 0.08	3.3 ± 0.3	4.5 ± 0.4	4.8 ± 0.3	0.15 ± 0.02
SK-BR-3	24	1.3 ± 0.05	2.3 ± 0.2	4.2 ± 0.3	6.7 ± 0.4	3.1 ± 0.2
	48	1.0 ± 0.03	1.6 ± 0.09	4.0 ± 0.2	3.0 ± 0.3	0.3 ± 0.02
	72	0.7 ± 0.01	1.5 ± 0.07	2.1 ± 0.2	2.6 ± 0.2	0.11 ± 0.01
MCF-12A	24	6.4 ± 0.2	10.9 ± 0.3	12.0 ± 0.6	13.0 ± 1.0	4.1 ± 0.2
	48	3.7 ± 0.4	6.2 ± 0.3	9.8 ± 0.5	7.3 ± 0.8	0.33 ± 0.02
	72	2.4 ± 0.1	4.8 ± 0.4	6.2 ± 0.4	6.0 ± 0.5	0.07 ± 0.01

Moreover, in the non-tumorigenic MCF-12A cells, the extract shows activity similar to that in MDA-MB-231 cells, with an IC50 value of 13.0 μg/mL (24 h of treatment; Figure 3A). Analysis of cell viability after treatment with individual compounds revealed that stizolin was the most effective across all studied breast cell types (tumor and non-tumorigenic). Moreover, izospiciformin revealed the least effect across all of the studied breast cell lines. Notably, the stizolin, stizolicin, and izospiciformin effectiveness was higher than the activity of *S. balsamita* extract. The analysis of cytotoxic activity of single compounds for 24 h in the panel of different molecular subtypes of breast cancer cells reveals that the most sensitive was the HER2-positive SK-BR-3 cell line, with the IC50 values 1.3 μg/mL, 2.3 μg/mL, and 4.2 μg/mL for stizolin, stizolicin, and izospiciformin, respectively. However, triple-negative breast cancer cells, represented by MDA-MB-231, show the least sensitivity, with IC50 values of 4.3 μg/mL for stizolin, 8.3 μg/mL for stizolicin, and 8.5 μg/mL for izospiciformin, respectively. Among all cell models, the ER + MCF7 cells showed medium sensitivity to both the extract and the studied single parthenolide derivatives.

Observed effectiveness of the studied compounds and the extract was similar and proportionally higher with prolonged exposure (48 and 72 h), with the least effectiveness of stizolin, stizolicin, and the extract against MCF7 breast cancer cells in the 72-h treatment interval (Figures 3B,C, respectively).

Notably, the extract and individual compounds showed weaker effects on non-tumorigenic MCF-12A breast cells across almost all tested time points, demonstrating the selectivity of the studied compounds relative to cancer cells and highlighting the varying cytotoxicities associated with their specific structures.

The selective cytotoxic activity of the studied compounds was expressed as a selectivity index (SI; Table 2), which was calculated for MCF-12A cells vs. the corresponding cancer cells (MCF7, MDA-MB-231, and SK-BR-3), using IC50 values. As demonstrated, all studied compounds (i.e., stizolin, stizolicin, izospiciformin, and extract) showed selectivity (with the range of 1.0–2.4) towards MCF7 and MDA-MB-231 breast cancer cells (referring to noncancer MCF12A cells). Interestingly, assessment of selectivity index in SK-BR-3 cells showed even higher values of SI (from 1.9 to 4.9). Doxorubicin was used as a positive cytotoxicity agent that is known to equally affect normal/noncancer and cancer cells (SI varied from 0.3 to 1.4).

**TABLE 2:** The calculated values of the selectivity index (SI) of the tested compounds and *Stizolophus balsamita* extract in the studied cells. SI≤1.00 in orange color (nonselective action), SI>1.00 in blue (selective action).

Cell line	Time [h]	SI (selectivity index)				
		Stizolin	Stizolicin	Izospiciformin	Extract	Doxorubicin
MCF7	24	2,4	2,4	1,3	1,3	0,3
	48	1,8	1,4	2,1	1,0	1,3
	72	1,2	1,3	1,6	1,0	1,2
MDA-MB-231	24	1,5	1,3	1,4	1,0	0,6
	48	1,0	1,1	1,4	1,2	1,4
	72	1,7	1,5	1,4	1,3	0,5
SK-BR-3	24	4,9	4,7	2,9	1,9	1,3
	48	3,7	3,9	2,5	2,4	1,1
	72	3,6	3,2	3,0	2,3	0,6

### 3.2 Colony-forming potential of studied tumor cell lines treated by stizolin, stizolicin, and izospiciformin

A colony formation assay was used to assess the genotoxic activity of compounds isolated from the *S. balsamita* extract in breast cancer cells MCF-7, MDA-MB-231, and SK-BR-3. Due to the weaker activity of the *S. balsamita* extract, only single compounds—stizolin, stizolicin, and izospiciformin—have been studied for affecting colony-forming potential. All the studied compounds were applied for 24 h at concentrations corresponding to 0.5×IC50, 1×IC50, and 1.5×IC50 values (Table 3). The 0.5×IC50, 1×IC50, and 1.5×IC50 values correspond to different degrees of cytotoxicity, from subcytotoxic (not yet causing significant detriment to the cell) to cytotoxic.

**TABLE 3:** The concentration range of stizolin, stizolicin, and izospiciformin applied in all experiments.

Cell line	Compound/Value	IC50 value for 24 h [µg/mL]		
		0.5 × IC50	1 × IC50	1.5 × IC50
MCF7	Stizolin	1.35	2.7	4.05
	Stizolicin	2.3	4.6	6.9
	Izospiciformin	4.65	9.3	13.95
MDA-MB-231	Stizolin	2.15	4.3	6.45
	Stizolicin	4.15	8.3	12.45
	Izospiciformin	4.25	8.5	12.75
SK-BR-3	Stizolin	0.65	1.3	1.95
	Stizolicin	1.15	2.3	3.45
	Izospiciformin	2.1	4.2	6.3

**FIGURE 4:**
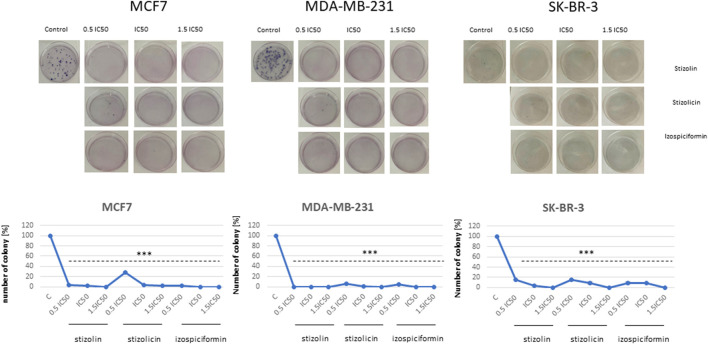
Influence of stizolin, stizolicin, and izospiciformin on the colony formation of MCF7, MDA-MB-231, and SK-BR-3 cells. The cells were plated at a density of 200 cells per well in a 6-well plate and allowed to adhere for 24 h. They were then treated with studied compounds in three different concentrations, corresponding to 0.5×IC50, 1×IC50, or 1.5×IC50 for 24 h. The experiment was repeated at least three times; x ± SD, p < 0.05. Statistically significant differences are shown versus control samples, ***p < 0.001.

This study revealed strong inhibitory effects on colony formation in all breast cancer cell lines following treatment with the studied compounds. The greatest effect was observed across all stizolin concentrations in all breast cancer cell lines and across the entire concentration range of all compounds in MDA-MB-231 cells. Moreover, at the 1.5×IC50 concentrations of all compounds completely reduced colony counts across all breast cancer cell lines studied (Figure 4).

### 3.3 Influence of stizolin, stizolicin, and izospiciformin on the cell cycle

**FIGURE 5:**
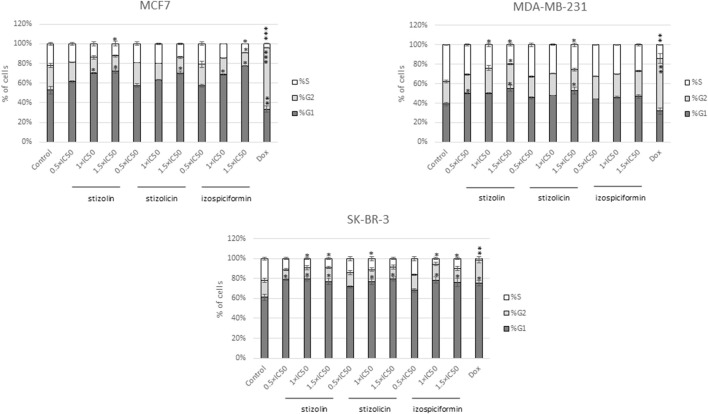
Cell cycle analysis of MCF7, MDA-MB-231, and SK-BR-3 cells treated with three different concentrations of PN derivatives for 24 h. Doxorubicin (Dox) at a concentration of 15 µM was used as the positive control for cell cycle alterations. Data from two independent experiments (in duplicates) are shown as mean ± SD; p < 0.05. A statistically significant difference is demonstrated versus control samples: *p < 0.05; **p < 0.005; ***p < 0.001.

Cell cycle analysis was performed after assessing viability and genotoxic compound activity in three breast cancer cell lines. All of the studied cell lines were treated with stizolin, stizolicin, or izospiciformin in the concentrations corresponding to 0.5×IC50, 1×IC50, and 1.5×IC50 for 24 h. Analysis of results revealed that in MCF7 cells stizolin (1×IC50 and 1.5 × IC50), stizolicin (1.5×IC50), and izospiciformin in the higher applied concentartions (IC50, and 1.5×IC50) increased population of cells in G1-phase (10%–20% increase) with reduction of the number of cells in the S- and G2-phases (up to 10% in both phases), relative to control cells (Figure 5).

Additionally, in MDA-MB-231, all studied PN derivatives at the highest applied concentration (1.5 × IC50) showed a similar effect: an increase in cell accumulation in the G1-phase, accompanied by a decrease in cells in the S-phase (around 15%). However, the most significant impact of studied compounds on the cell cycle distribution was observed in SK-BR-3 cells, with the increase of G1 phase (around 15%) and decrease of cell number in the S-phase after treatment of stizolin in the whole concentration range. Similarly, higher concentrations of stizolicin and izospiciformin (1 × IC50, and 1.5 × IC50) provoked a similar effect (around 10%–15% depending on compound concentration).

### 3.4 Analysis of proapoptotic activity of studied compounds in breast cancer cells

**FIGURE 6:**
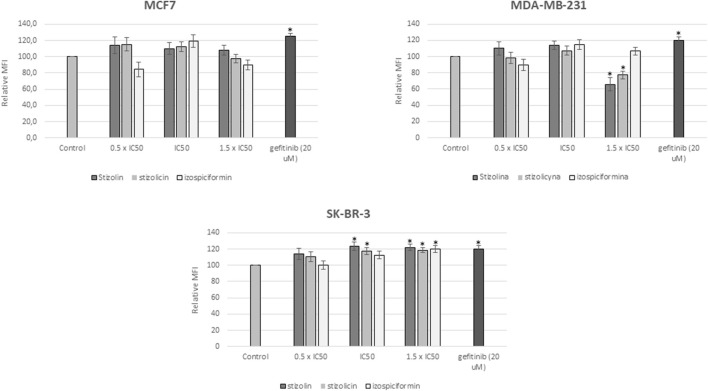
Evaluation of apoptosis induction by PN derivatives. MCF7, MDA-MB-231, and SK-BR-3 cells were treated with the studied compounds at 0.5×IC50, 1×IC50, or 1.5×IC50, respectively, followed by propidium iodide (PI) staining and mean fluorescence intensity (MFI) assessment. Bars represent the relative MFI of PI staining in the indicated samples compared with their corresponding controls. Gefitinib (Gef, 20 µM) was used as a positive control for apoptosis. The mean of three independent experiments (x±SD) is shown, with p < 0.05 as the cut-off for statistical significance.

To verify whether the cytotoxic effect observed in MTT and clonogenic assays is associated with cell elimination via apoptosis, cells were stained with propidium iodide after 24 h of compound treatment. All of the studied breast cancer cell lines were treated with stizolin, stizolicin, or izospiciformin at concentration values of 0.5–1.5×IC50. As a positive apoptotic control, gefitinib at a concentration of 20 µM has been used. Analysis of mean fluorescence intensity (MFI) revealed that the studied compounds did not induce apoptosis in MCF7 and MDA-MB-231 cells. Nevertheless, in MDA-MB-231 cells treated with the higher concentration (i.e. 1.5×IC50) of stizolin and stizolicin, a decrease in MFI derived from PI staining has been observed. However, in HER2-positive breast cancer cells, an increase in apoptosis has been observed after 24 h of treatment with all studied compounds, especially at 1×IC50 and 1.5×IC50 (15%–25%) (Figure 6).

**FIGURE 7:**
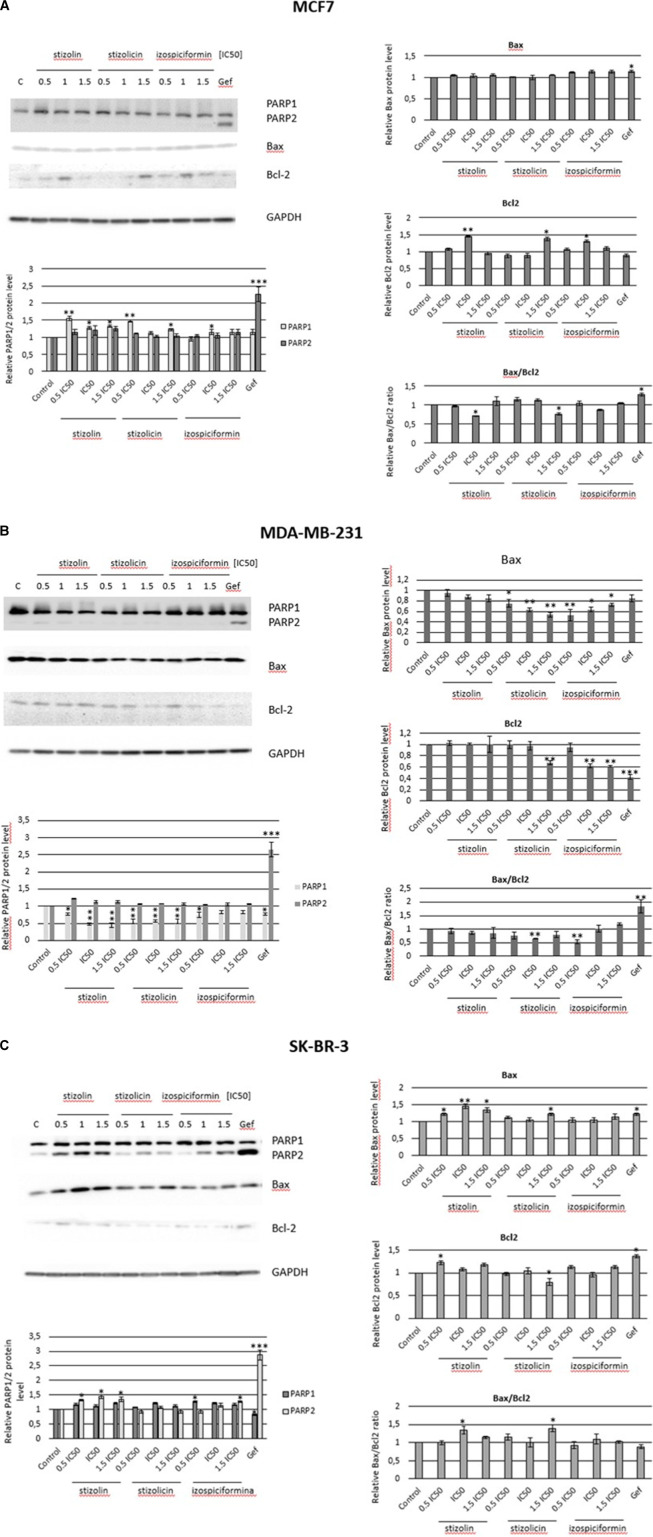
Assessment of the proapoptotic potential of PN derivatives. MCF7 **(A)**, MDA-MB-231 **(B)**, and SK-BR-3 **(C)** cells were treated with the studied compounds at 0.5×IC50, 1×IC50, or 1.5×IC50, followed by immunodetection. The immunoidentification of target proteins (PARP1/2, Bax, Bcl2) was subjected to densitometry analysis, normalized to GAPDH. Gefitinib (Gef, 20 µM) was used as a positive control for apoptosis. The mean value of two experiments ±SD is shown. A statistically significant difference is demonstrated versus control samples: *, p < 0.05; **, p < 0.005; ***, p < 0.001.

To verify the proapoptotic potential of the studied compounds in cells representing different breast cancer subtypes, Western blot and immunoidentification were performed to analyze PARP1/2, Bax, and Bcl-2 protein levels (Figure 7). In MCF7 cells, we observed a reduced Bax/Bcl2 ratio after treating cells with stizolin (1×IC50) and stizolicin (1.5 × IC50) (Figure 7A). Additionally, a similar effect was observed in MDA-MB-231 cells, where the Bax/Bcl2 ratio was decreased in cells treated with stizolicin (1×IC50) and izospiciformin (0.5×IC50) (Figure 7B), which suggests antiapoptotic activity of these PN derivatives in ER-positive and basal subtypes of breast cancer cells.

However, apoptosis has been observed in SK-BR-3, HER2-positive breast cancer cells, following treatment with stizolin or stizolicin, as assessed by PARP1/2 protein levels and the Bax/Bcl2 ratio. This study revealed a 40% increase in apoptosis at the concentration of stizolin corresponding to the 1×IC50, as well as at the highest concentration of stizolicin (1.5×IC50) (Figure 7C). These results corresponded to the PI staining-based apoptosis detection (Figure 6).

### 3.5 Autophagy detection (monodansylcadaverine (MDC) staining)

Assessment of apoptosis induced by the studied compounds revealed selective proapoptotic activity in SK-BR-3 cells. However, the MTT assay and clonogenic test showed high cytotoxicity of the studied compounds across all studied breast cancer cells; thus, it was interesting to investigate whether PN derivatives in breast cancer cells induce other cell death pathways and/or metabolic alterations. Therefore, one of the alternative mechanism responsible for cell fate i.e., autophagy was evaluated.

**FIGURE 8:**
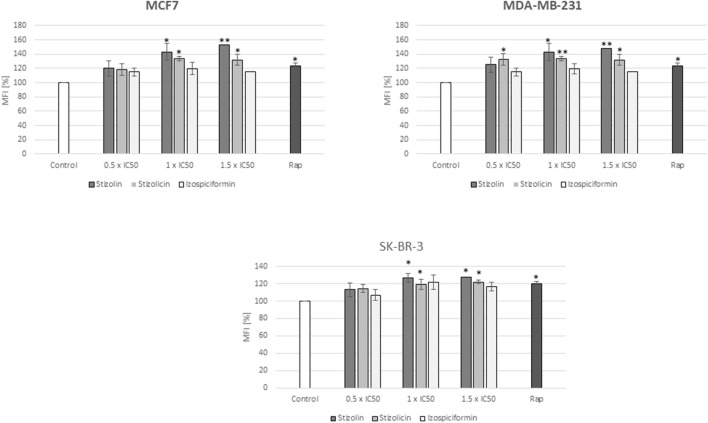
Evaluation of autophagy induction by PN derivatives. MCF7, MDA-MB-231, and SK-BR-3 cells were treated with the studied compounds for 24 h at 0.5×IC50, 1×IC50, or 1.5×IC50, respectively, followed by monodansylcadaverine (MDC) staining to determine mean fluorescence intensity (MFI). Bars represent the relative MFI of MDC staining in the indicated samples versus the corresponding controls. Rapamycin (Rap, 50 nM) was used as a positive control for autophagy. The mean of three independent experiments is shown with p < 0.05 calculated from paired data. x±SD, p < 0.05 (A). Statistically significant differences versus control samples are shown: *p < 0.05; **p < 0.005.

First, monodansylcadaverin (MDC) staining, a screening assay for visualizing and quantifying autophagy, has been performed. MDC stains acidic structures in cells, such as lysosomes and autolysosomes, where autophagy also occurs (Murugan and Amaravadi, 2016). The mean fluorescence intensity analysis from MDC cells stained after compound treatment revealed that stizolin, at concentrations corresponding to 1 x IC50 and 1.5 × IC50 values, induces autophagy in all breast cancer cell lines (30%–50% increase relative to control, depending on cell line). Moreover, across all studied breast cancer cell models, stizolicin also exhibited proautophagic activity at higher applied compound concentrations (1×IC50 and 1.5×IC50) (20%–30%). However, the izospiciformin did not significantly alter MDC cell staining in MCF7, MDA-MB-231, or SK-BR-3 cells, compared to control, untreated cells (Figure 8).

To confirm the proautophagic properties of the studied compounds in different breast cancer cell lines, Western blot and immunoidentification analyses of autophagy marker proteins (MAPLC3, p62/SQSTM, mTOR) were performed. MAPLC3 (LC3) is currently the most reliable marker of autophagosomes, as LC3-II levels reflect the number of autophagosomes and autophagy-related structures. In its soluble form, LC3-I is a precursor protein present in the cytoplasm. After autophagy is initiated, LC3-I is conjugated to phosphatidylethanolamine (PE), forming the membrane-bound form LC3-II. LC3-II is recruited to the autophagosome membrane, and its level correlates with the number of autophagosomes formed. A higher ratio of LC3II to LC3I (LC3II/LC3I) indicates that more LC3I has been processed, which is a direct indicator of autophagosome formation (Saito et al., 2026).

**FIGURE 9:**
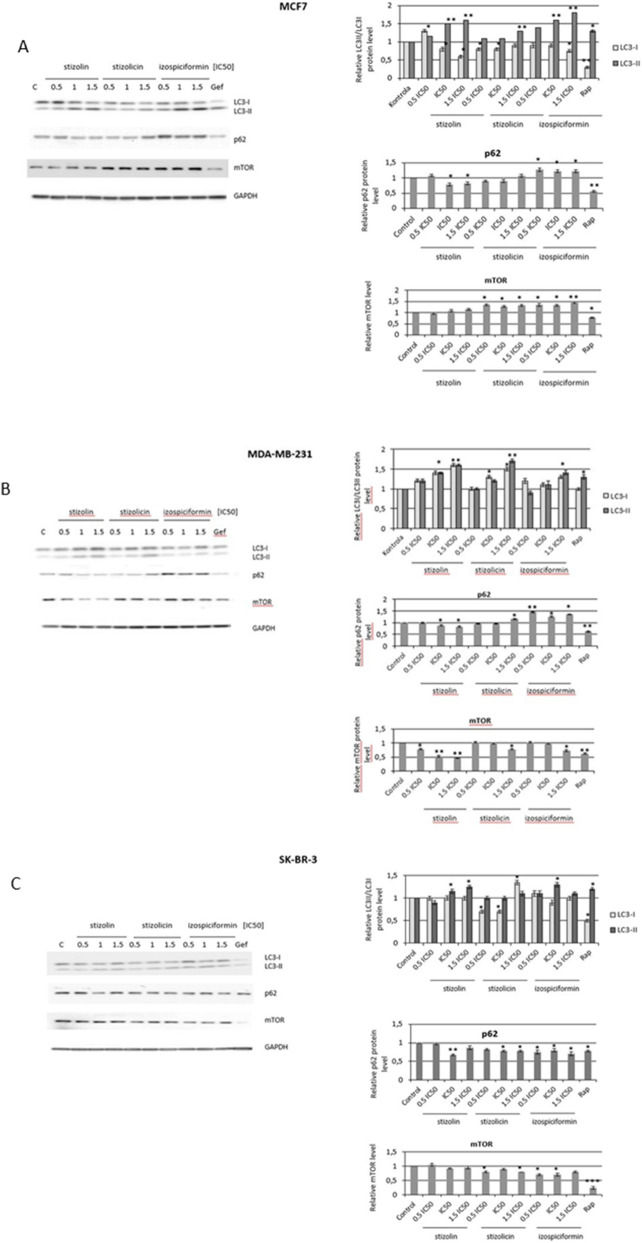
Evaluation of autophagy induction by PN derivatives in breast cancer cells. MCF7 **(A)**, MDA-MB-231 **(B)**, and SK-BR-3 **(C)** cells were treated with the studied compounds at 0.5×, 1×, or 1.5× IC50, respectively, for 24 h. Rapamycin (Rap, 50 nM) was used as a positive control for autophagy. The immunoidentification of marker proteins (LC3II/LC3I, p62, mTOR) was accompanied by densitometry analysis, normalized to GAPDH. The mean of two independent experiments ±SD is shown, with p values calculated from paired data. Statistically significant differences versus control samples are shown: *, p < 0.05; **, p < 0.005; ***, p < 0.001.

p62/SQSTM degradation is another commonly used marker for monitoring autophagic activity, as p62 binds directly to LC3 and is selectively degraded by autophagy. Autophagy induction converts LC3-I to LC3-II and increases LC3-II levels, accompanied by a concomitant decrease in p62 levels (Mandic et al., 2024). Another autophagy modulator, mTOR, has many biological functions; however, in the context of autophagy, it acts as a negative regulator (Pan et al., 2023). As a positive autophagy control, rapamycin was used at 50 nM for 24 h. Treatment of ER-positive cells (MCF7) with the studied parthenolid derivatives revealed that stizolin, at concentrations corresponding to 1×IC50 and 1.5 × IC50, exhibited proautophagic activity (increase in the LC3II/LC3I ratio and decrease in p62). However, izospiciformin, at higher compound concentrations (1×IC50 and 1.5×IC50), inhibits autophagy in MCF7 cells, that is manifested by a reduction in the LC3II/LC3I ratio and an increase in mTOR and p62 protein levels (Figure 9A), without any significant effect caused by stizolicin. Moreover, in the basal subtype, the MDA-MB-231 breast cancer cell line, stizolin significantly increased autophagy at concentrations corresponding to the IC50 and 1.5 × IC50. However, in the basal subtype of breast cancer, stizolicin and izospiciformin show no effect on autophagy markers (Figure 9B). Additionally, in HER-2 positive, SK-BR-3 breast cancer cells, izospicifromin and stizolin increased the LC3II/LC3I ratio (IC50), with a decrease of mTOR and p62 protein levels (after treatment with izospicifromin of approximately 20%–30%, and for stizolin approximately 10%–30%). However, in this cell line, stizolicin did not provoke autophagy (Figure 9C).

## 4 Discussion

Breast cancer is a heterogeneous disease that constitutes a serious therapeutic challenge. Selecting the appropriate drug is crucial due to the diverse responses of different molecular subtypes of breast cancer. Targeted therapy has enabled the use of individualized strategies that minimize side effects and improve patient outcomes. However, challenges remain, particularly in understanding and addressing resistance mechanisms and in developing more effective therapies (Carvalho et al., 2025).

Parthenolide and its derivatives are known to attenuate pathogenicity and increase the sensitivity of various cancer types to chemotherapy or radiotherapy. It is well documented that PN exhibits cytotoxicity against multiple cancer types and is well tolerated in humans (Zarei et al., 2025). Parthenolide is characterized by its ability to induce cancer cell death while sparing normal cells. Guzman L.M. and collaborators demonstrated that parthenolide induced apoptosis in myeloid leukemia (AML) cells without affecting normal hematopoietic cells (Guzman et al., 2007). Moreover, in preclinical studies parthenolide was effective treating relapsed leukemia associated with multidrug resistance, which is driven by leukemia stem cells (Kim et al., 2021; Zarei et al., 2025). The limitations of parthenolide use are repeatedly emphasized due to its low bioavailability and stability. Therefore, much attention has been paid to the anticancer potential of its derivatives, such as dimethylaminoparthenolide (DMAPT), which have been the subject of preclinical *in vitro* and *in vivo* studies. This derivative is characterized by higher water solubility and oral bioavailability (Guzman et al., 2007). DMAPT, like parthenolide, induces cancer cell death, has documented radiosensitivity properties against prostate cancer cell lines and breast cancer stem cells, and demonstrates radioprotective properties in normal cells (Mendonca et al., 2017; Carlisi et al., 2016). Notably, parthenolide and its derivatives have also been shown to exhibit anticancer properties in breast cancer. Several studies have demonstrated cytotoxic effects of parthenolide and its derivatives in the basal subtype of breast cancer, specifically in MDA-MB-231 cells and the estrogen receptor-positive MCF7 cells. Both cell lines treated with PN demonstrated inhibition of proliferation (the IC50 value of PN for MCF is 2.5 μg/mL, and for MDA-MB-231, the IC50 = 4.4 μg/mL, data from 48-h treatment experiment (Bampton et al., 2005; Al-Fatlawi et al., 2014). Another study demonstrated reductions in MDA-MB-231 cell viability of over 60% and 70% after 16 h of treatment with 6.2 μg/mL parthenolide and 7.3 μg/mL DMAPT, respectively. Moreover, the PN and its derivatives stimulate the production of reactive oxygen species, which initiate a cascade of events leading to cell destruction (Jorge et al., 2023).

The extract of *S. balsamita* leaves assessed in our study is a mixture of seven sesquiterpene lactones: balsamin, stizolin, stizolicin, 9α-hydroxyparthenolide, izospiciformin, 8α-E-(4′-hydroxy)-senecioyloxy-9α-hydroxyparthenolide, and 11βH,13-dihydrostizolicin (Nawrot et al., 2019a). The emphasis should be on the absence of parthenolide in the studied extract. The cytotoxic effect of selected, dominant compounds was stronger than that observed with the extract. This is an interesting observation, suggesting a diminishing rather than an additive effect of these sesquiterpenes. Additionally, another significant information is that the content of derivatives in *S. balsamita* leaves remains unchanged over time, and their properties remain stable (tested over a period of 3 years) (Nawrot et al., 2019b). This is a crucial parameter in the context of the PN instability.

Sesquiterpen lactones, like parthenolide and its derivatives evaluated in this study, have three common elements of structure in their skeleton: a 4,5-epoxide and a lactone ring conjugated to an exomethylene (Nawrot et al., 2019b). The presence of an epoxide group, through its ability to covalently bind to nucleophilic centers of proteins and nucleic acids, inhibits enzyme activity, induces cell cycle arrest, and activates apoptosis (Rodrigues et al., 2019). Additionally, the presence of a lactone in the chemical structure of compounds is associated with antimicrobial, antifungal, antiviral, antimigraine, anti-inflammatory, and antitumor activities (Nawrot et al., 2021; Nawrot et al., 2019b; Zhao et al., 2025; Mazur and Masłowiec, 2022).

In studies performed by Nawrot et al., stizolin, stizolicin, and izospiciformin exhibited anti-inflammatory and antiserotonin effects by inhibiting 5-HT release from platelets. These PN derivatives were more effective than parthenolide, with isospiciformin demonstrating the strongest effect (Nawrot et al., 2020). Stizolicin also exhibits remarkable cytotoxic and antiparasitic activity (Nawrot et al., 2021).

Among all of the studied PN derivatives, stizolin has the most similar chemical structure to parthenolide. The crucial difference is the presence of a hydroxyl group at position C8. Available data indicate that the presence of a hydroxyl group in the structure, particularly in the C8 position of the PN structure of compounds, enhances their solubility in water and is associated with the purple color of parthenolide (Nawrot et al., 2019a; Nawrot et al., 2020; Wyganowska-Światkowska et al., 2020; Cramer et al., 2019). However, stizolicin, compared with parthenolide, possesses an additional side chain, 4-hydroxy-2-hydroxymethyl-2E-butenoyl, which supports its antifungal, anticancer, antiinflammatory, and antioxidant properties. They also improve water solubility and increase interactions with biological targets (Klecker and Nair, 2017; Rodrigues et al., 2019; Siswina et al., 2023). The third of the studied PN derivatives, izospiciformin, in comparison to PN, has a lactone ring in a different position: C7-C8 (PN has a lactone ring in position C6-C7), and an additional hydroxyl group at C6 (Figure 2). The location of the lactone ring and the hydroxyl group appear to play a crucial role in the distinct and selective biological activities of stizolin and izospiciformin in the studied cell lines.

To verify the toxicity of studied PN derivatives and assess the viability and reproductive capacity of compounds across different molecular subtypes of breast cancer cell lines, MTT and colony-formation assays have been performed. This solution is optimal to study if the drug will have short-term toxicity in the culture, but it may also have residual toxicity, observed in long-term conditions. The cells may not have died in the short term (24, 48 h), but the drug had residual toxicity, manifested as loss of clonogenicity in these cells (Forgie et al., 2024). Consequently, clonogenic assay (Figure 4) showed genotoxic potential of studied compounds. Altogether, in our study, we found that stizolin exhibited significant cytotoxicity at short-term exposure, while all studied PN derivatives led to inhibition of the cells’ reproductive capacity measured in clonogenic assay. Thus, the studies performed in MCF7, MDA-MB-231, SK-BR-3, and MCF-12A cells showed high biological activity of stizolin, stizolicin, izospiciformin or Stizolophus balsamita extract. Consequently, selectivity index (SI) was calculated for breast cancer cells referring to noncancer MCF-12A cells (Table 2) that suggested higher biological activity of the compounds in cancer than in normal cells. Comparison of IC50 values for studied compounds is a well-established approach when cancer cells’ specificity is evaluated (Tronina et al., 2023). Evaluation was performed for three time intervals i.e. 24, 48, and 72 h and the results demonstrated high consistency regarding predominant diminish of breast cancer cell viability compared to noncancer breast cells.

Although MTT altogether with clonogenic assay demonstrated high biological potential of studied compounds they could not reveal the mechanism of this viability impairment. While MTT shows general metabolic activity alterations (due to redox conditions imbalance) the clonogenic assay shows the ability of studied compounds to affect proliferation potential and colony formation potential. This in turn can correspond to different cell death types induction, including mitotic catastrophe, apoptosis, necrosis, cellular senescence or programmed necrosis (parthanatos) (Bravo-San Pedro et al., 2021; Chang et al., 2021). Consequently further investigation aimed identification of the mechanism of action of studied compounds. These experiments involved the influence of the studied compounds on cell death and apoptosis, which are desired outcomes of an anticancer therapeutic strategy. The available data demonstrate proapoptotic properties of parthenolide and its derivative in human colorectal cancer cells and cholangiocarcinoma which were manifested by alterations in Bax and Bcl-2 (Santos et al., 2022; Carlisi et al., 2022). Interestingly, in our study, parthenolide derivatives did not provoke apoptosis in MCF7 or MDA-MB-231 cells, but they selectively induced apoptosis in HER2-positive breast cancer cells SK-BR-3. Due to overexpression of the epidermal growth factor receptor, these cells exhibit an aggressive phenotype, proliferate more rapidly, may metastasize, have a poorer prognosis, and do not respond to therapy (Cheng, 2024). The observed effect of parthenolide derivatives is consequently highly desirable in the context of this type of malignant tumor. The studies also showed higher effectiveness of action, especially of stizolin (IC50 in the range 1.3–4.3 μg/mL for 24 h, depending on breast cancer cell line). There is currently no available data on the biological activity of PN in SK-BR-3 cells; however, our results showed that stizolin had the greatest cytotoxic effect in those cells. According to available data on the cytotoxicity of parthenolide in MCF7 and MDA-MB-231 (Santos et al., 2022), it is worth noting that stizolin exhibits approximately 20% higher cytotoxicity than the maternal compound, PN, in both ER-positive and basal breast cancer cell lines, respectively.

Next, evaluation of PARP1 and PARP2 accumulation was performed. PARP1 and PARP2 are nuclear enzymes crucial for DNA damage detection and repair, primarily promoting cell survival by maintaining genomic stability. Under mild damage, they facilitate repair, but excessive activation by high-stress conditions leads to PARP accumulation, leading to energy depletion and programmed necrosis (parthanatos) (Huang et al., 2022). PARP1 is the major player, while PARP2 contributes to a lesser extent but can partially compensate for the loss of PARP1. In MCF7 cells exposed to studied compounds a significant increase of PARP1 was observed after treatment with stizolin (whole range), stizolicin (0.5×IC50 and 1.5×IC50), and izospiciformin (1×IC50). It corresponded with Bcl-2 accumulation which suggested antiapoptotic effect or a response that implies targetable dependency and possible success of Bcl-2-based anti-apoptotic approach. In MDA-MB-231 an opposite effect was observed i.e., significant decrease of PARP1 that was accompanied by Bax decrease which again suggests antiapoptotic effect of studied compounds. In SK-BR-3 PARP1 and PARP2 increase accompanied by Bax accumulation was observed that implied proapoptotic effect of studied compounds. However, since there was no consistency in pro- or antiapoptotic signaling and cytotoxicity/clonogenic assay further experiments were designed to evaluate potential contribution of studied compounds to another death type–autophagy.

Autophagy is a process of dual nature. It can both provide cell death or survival. Consequently, autophagy modulation requires a strictly defined treatment strategy to eliminate the risk of possible adverse effects (Huang et al., 2025). For this reason assessment of autophagy efficacy is based on identification of several markers, i.e., monodansylcadaverine (MDC) staining followed by LC3II/I, p62 and mTOR assessment is a gold standard approach. LC3 turnover reflects the level autophagosomes accumulation of is due to increased induction or decreased degradation (Castillo et al., 2013). Additionally we performed monodansylcadaverine labeling which specifically stains autophagosomes in mammals (Lu and Djabali, 2018). In mammalian cells, the specificity of MDC staining is derived both from ion trapping, as autophagosomes are known to be acidic compartments, and interaction with lipid molecules found in high concentration in autophagosomes (Sassi et al., 2019).

Our cytotoxicity analysis, confirmed by colony-formation assay and cell cycle analysis, demonstrated that the studied compounds, especially stizolin, decrease cell viability and exhibit anti-proliferative properties; thus, the induction of autophagy in this context has a destructive effect on cancer cells. Interestingly, in the case of the autophagy process, it was revealed that izospiciformin shows anti-autophagic abilities, but only in MCF7 breast cancer cells (the only one from the studied breast cancer cell lines expressing estrogen and progesterone receptors). In all studied cell lines, this compound showed the highest IC50 values, 2-3-fold higher than stizolin, thus lower cytotoxicity. Moreover, in the basal subtype (MDA-MB-231 cells), this derivative showed an antiapoptotic effect, suggesting weaker biological activity in this subtype of breast cancer. However, in HER-2-positive BC, SK-BR-3 cells, this PN derivative had the highest cytotoxicity among all studied PN derivatives (2-fold higher at 24 h of treatment) and also induced autophagy. This emphasizes the selectivity of studied compounds and the diverse mechanisms of biological activity in different molecular subtypes of breast cancer.

Our results demonstrate compound-dependent activity and selectivity of the studied natural parthenolide derivatives in different molecular subtypes of breast cancer. This selectivity is also associated with a weaker cytotoxic effect against the non-cancerous MCF-12A breast cells. Our preliminary studies revealed interesting findings and encourage further mechanistic investigations, for the development of new compounds or strategies dedicated to specific molecular subtypes of breast cancer patients. Additionally, some possible combination/adjuvant approach could be applied based on such studies. Referring to other, natural-derived compounds, it is worth emphasizing that natural products such as curcumin, resveratrol, and quercetin show promise as adjuvant treatments for different breast cancer subtypes, enhancing the efficacy of conventional therapies (e.g., paclitaxel, doxorubicin) and diminishing side effects. These compounds can reverse multidrug resistance, inhibit tumor growth, and decrease toxicity to normal tissues. However, clinical evidence is still evolving, and plant-based drug interactions must be carefully monitored (Kobakova et al., 2025). Although demonstrated results refer to *in vitro* conditions, we believe that they indicate new potential pathways that can be addressed in the context of an anticancer strategy. It will definitely be interesting to examine the properties of these compounds in combination with cancer drugs and in 3D cell systems. Another aim would be to evaluate the potential of these natural compounds in targeting cancer stem cells, which constitute another therapeutic challenges especially since they are controlled by HER2-associated pathways (Qiu et al., 2021).

## 5 Conclusion

Conducted research on a panel of different breast cancer cell lines, representing various molecular subtypes of breast cancer revealed different biological activity of parthenolide derivatives, stizolin, stizolicin, and izospiciformin. Considering the difficulties of PN bioavailability, the presence of a hydroxyl group in the stizolin structure appears to address this issue, particularly in HER-2-positive breast cancer cells. Notably, the studied compounds demonstrated selectivity for cancer cells and showed higher activity when used alone than in combination with other compounds in the extract of *S. balsamita*. Our results, although preliminary, suggest a direction for designing drugs with higher biological activity across different molecular subtypes of breast cancer cells.

## Data Availability

The original contributions presented in the study are included in the article/supplementary material, further inquiries can be directed to the corresponding author.

## References

[B1] Al-Fatlawi A. Al-Fatlawi A. Rahisuddin I. Ahmad A. (2014). Effect of parthenolide on growth and apoptosis regulatory genes of human cancer cell lines. Pharm. Biol. 53 (1), 104–109. 10.3109/13880209.2014.911919 25289524

[B2] Bampton T. E. W. Goemans G. C. Niranjan D. Mizushima N. Tolkovsky A. M. (2005). The dynamics of autophagy visualised in live cells: from autophagosome formation to fusion with endo/lysosomes. Autophagy 1 (1), 23–36. 10.4161/auto.1.1.1495 16874023

[B3] Berdan C. A. Ho R. Lehtola H. S. To M. Hu X. Huffman T. R. (2019). Parthenolide covalently targets and inhibits focal adhesion kinase in breast cancer cells. Cell Chem. Biol. 26 (7), 1027–1035.e22. 10.1016/j.chembiol.2019.03.016 31080076 PMC6756182

[B4] Bravo-San Pedro J. M. Kepp O. Sauvat A. Rello-Varona S. Kroemer G. Senovilla L. (2021). Clonogenic assays to detect cell fate in mitotic catastrophe. Methods Mol. Biol. 2267, 227–239. 10.1007/978-1-0716-1217-0_16 33786796

[B5] Carlisi D. Buttitta G. Di Fiore R. Scerri C. Drago-Ferrante R. Vento R. (2016). Parthenolide and DMAPT exert cytotoxic effects on breast cancer stem-like cells by inducing oxidative stress, mitochondrial dysfunction and necrosis. Cell Death Dis. 7, e2194. 10.1038/cddis.2016.94 27077810 PMC4855673

[B6] Carlisi D. Lauricella M. D’Anneo A. De Blasio A. Celesia A. Pratelli G. (2022). Parthenolide and its soluble analogues: multitasking compounds with Antitumor properties. Biomedicines 10 (2), 514. 10.3390/biomedicines10020514 35203723 PMC8962426

[B7] Carvalho E. Canberk S. Schmitt F. Vale N. (2025). Molecular subtypes and mechanisms of breast cancer: precision medicine approaches for targeted therapies. Cancers 17 (7), 1102. 10.3390/cancers17071102 40227634 PMC11987866

[B8] Casaubon J. T. Kashyap S. Regan J. P. (2023). BRCA1 and BRCA2 Mutations. Treasure Island (FL): StatPearls. Available online at: www.ncbi.nlm.nih.gov/books/NBK470239 (Accessed April 06, 2026). 29262038

[B9] Castillo K. Valenzuela V. Matus S. Nassif M. Oñate M. Fuentealba Y. (2013). Measurement of autophagy flux in the nervous system *in vivo* . Cell Death Dis. 4, e917. 10.1038/cddis.2013.421 24232093 PMC3847309

[B10] Chang D. S. Lasley F. D. Das I. J. Mendonca M. S. Dynlacht J. R. (2021). “Modes of cell death and survival assays,” in Basic radiotherapy physics and biology. Cham: Springer. 10.1007/978-3-030-61899-5_22

[B11] Cheng X. (2024). A comprehensive review of HER2 in cancer biology and therapeutics. Genes (Basel) 15 (7), 903. 10.3390/genes15070903 39062682 PMC11275319

[B12] Cohen S. Y. Stoll C. R. Anandarajah A. Doering M. Colditz G. A. (2023). Modifiable risk factors in women at high risk of breast cancer: a systematic review. Breast Cancer Res. 25 (1), 45. 10.1186/s13058-023-01636-1 37095519 PMC10123992

[B13] Cramer J. Sager C. P. Ernst B. (2019). Hydroxyl groups in synthetic and natural-product-derived therapeutics: a perspective on a Common Functional Group. J. Med. Chem. 62, 20–8930. 10.1021/acs.jmedchem.9b00179 31083946

[B14] Denda Y. Matsuo Y. Sugita S. Eguchi Y. Nonoyama K. Murase H. (2024). The natural product parthenolide inhibits both angiogenesis and invasiveness and improves gemcitabine resistance by suppressing nuclear factor κB activation in pancreatic cancer cell lines. Nutrients 16 (5), 705. 10.3390/nu16050705 38474833 PMC10934733

[B15] Forgie B. N. Prakash R. Goyeneche A. A. Telleria C. M. (2024). Vitality, viability, long-term clonogenic survival, cytotoxicity, cytostasis and lethality: what do they mean when testing new investigational oncology drugs? Discov. Oncol. 15 (1), 5. 10.1007/s12672-023-00857-2 38180601 PMC10769964

[B16] García-Sancha N. Corchado-Cobos R. Pérez-Losada J. (2025). Understanding susceptibility to breast cancer: from risk factors to prevention strategies. Int. J. Mol. Sci. 26, 2993. 10.3390/ijms26072993 40243654 PMC11988588

[B17] Guzman L. M. Rossi R. M. Neelakantan S. Li X. Corbett C. A. Hassane D. C. (2007). An orally bioavailable parthenolide analog selectively eradicates acute myelogenous leukemia stem and progenitor cells. Blood 110 (13), 4427–4435. 10.1182/blood-2007-05-090621 17804695 PMC2234793

[B18] Huang P. Chen G. Jin W. Mao K. Wan H. He Y. (2022). Molecular mechanisms of parthanatos and its role in diverse diseases. Int. J. Mol. Sci. 23 (13), 7292. 10.3390/ijms23137292 35806303 PMC9266317

[B19] Huang X. Yan H. Xu Z. Yang B. Luo P. He Q. (2025). The inducible role of autophagy in cell death: emerging evidence and future perspectives. Cell Commun. Signal. 23, 151. 10.1186/s12964-025-02135-w 40140912 PMC11948861

[B20] Jorge J. Neves J. Alves R. Geraldes C. Gonçalves A. C. Sarmento-Ribeiro A. B. (2023). Parthenolide induces ROS-Mediated apoptosis in lymphoid malignancies. Int. J. Mol. Sci. 24 (11), 9167. 10.3390/ijms24119167 37298119 PMC10252365

[B21] Kim J. Harper A. McCormack V. Sung H. Houssami N. Morgan E. (2025). Global patterns and trends in breast cancer incidence and mortality across 185 countries. Nat. Med. 31, 1154–1162. 10.1038/s41591-025-03502-3 39994475

[B58] Kim Y. Sengupta S. Sim T. (2021). Natural and Synthetic Lactones Possessing Antitumor Activities. Int. J. Mol. Sci. 22, 1052. 10.3390/ijms22031052 33494352 PMC7865919

[B22] Klecker C. Nair L. S. (2017). “Chapter 13 - matrix chemistry controlling stem cell behavior,” in Biology and engineering of stem cell niches. Academic Press, 195–213. 10.1016/B978-0-12-802734-9.00013-5

[B23] Kobakova Y. Moneva-Sakelarieva M. Konstantinov S. Momekov G. Ivanova S. A. Atanasova V. (2025). Natural products as part of triple negative breast cancer. Pharmacia 72, 1–11. 10.3897/pharmacia.72.e143465

[B24] Lakhani S. R. Ellis I. O. Schnitt S. J. Tan P. H. van de Vijver M. J. (2012). World Health Organization. WHO classification of tumours. WHO classification of tumours of the breast. 4th Edition, 4. IARC Publications. Available online at: https://publications.iarc.who.int/Book-And-Report-Series/Who-Classification-Of-Tumours/WHO-Classification-Of-Tumours-Of-The-Breast-2012 (Accessed January 14, 2026).

[B25] Li J. Li X. Liu H. (2025). Sesquiterpene lactones and cancer: new insight into antitumor and anti-inflammatory effects of par-thenolide-derived Dimethylaminomicheliolide and micheliolide. Front. Pharmacol. 16, 1551115. 10.3389/fphar.2025.1551115 40051564 PMC11882563

[B26] Lisiak N. Paszel-Jaworska A. Bednarczyk-Cwynar B. Zaprutko L. Kaczmarek M. Rybczyńska M. (2014). Methyl 3-hydroxyimino-11-oxoolean-12-en-28-oate (HIMOXOL), a synthetic oleanolic acid derivative, induces both apoptosis and autophagy in MDA-MB-231 breast cancer cells. Chem.-Biol. Interact. 208, 47–57. 10.1016/j.cbi.2013.11.009 24291674

[B27] Lisiak N. Dzikowska P. Wisniewska U. Kaczmarek M. Bednarczyk-Cwynar B. Zaprutko L. (2023). Biological activity of oleanolic acid derivatives HIMOXOL and Br-HIMOLID in breast cancer cells is mediated by ER and EGFR. Int. J. Mol. Sci. 24 (6), 5099. 10.3390/ijms24065099 36982173 PMC10048893

[B28] Liu J. Cui M. Wang Y. Wang J. (2023). Trends in parthenolide research over the past two decades: a bibliometric analysis. Heliyon 9 (7), e17843. 10.1016/j.heliyon.2023.e17843 37483705 PMC10362189

[B29] Liu X. Z. Tai Y. Hou Y. B. Cao S. Han J. Li M. (2024). Parthenolide inhibits synthesis and promotes degradation of programmed cell death ligand 1 and enhances T cell tumor-killing activity. J. Agric. Food Chem. 72, 38–21029. 10.1021/acs.jafc.4c04916 39264009

[B30] Lu X. Djabali K. (2018). Autophagic removal of farnesylated carboxy-terminal lamin peptides. Cells 7 (4), 33. 10.3390/cells7040033 29690642 PMC5946110

[B31] Mandic M. Paunovic V. Vucicevic L. Kosic M. Mijatovic S. Trajkovic V. (2024). No energy, no autophagy—Mechanisms and therapeutic implications of autophagic response energy requirements. J. Cell. Physiol. 239, e31366. 10.1002/jcp.31366 38958520

[B32] Mazur M. Masłowiec D. (2022). Antimicrobial activity of lactones. Antibiotics 11 (10), 1327. 10.3390/antibiotics11101327 36289985 PMC9598898

[B33] Mendonca M. S. Turchan W. T. Alpuche M. E. Watson C. N. Estabrook N. C. Chin-Sinex H. (2017). DMAPT inhibits NF-κB activity and increases sensitivity of prostate cancer cells to X-rays *in vitro* and in tumor xenografts *in vivo* . Free Radic. Biol. Med. 112, 318–326. 10.106/j.freeradbiomed.2017.08.001 28782644 PMC6322835

[B34] Michalak M. Stryjecka M. Żarnowiec P. Zagórska-Dziok M. Kiełtyka-Dadasiewicz A. (2024). Chemical composition of extracts from various parts of feverfew (Tanacetum parthenium L.) and their antioxidant, protective, and antimicrobial activities. Int. J. Mol. Sci. 25 (22), 12179. 10.3390/ijms252212179 39596244 PMC11594288

[B35] Murugan S. Amaravadi R. K. (2016). Methods for studying autophagy within the tumor microenvironment. Adv. Exp. Med. Biol. 899, 145–166. 10.1007/978-3-319-26666-4_9 27325266 PMC5451257

[B36] Nawrot J. Budzianowski J. Nowak G. (2019a). Phytochemical profiles of the leaves of Stizolophus balsamita and Psephellus sibi-ricus and their chemotaxonomic implications. Phytochemistry 159, 172–178. 10.1016/j.phytochem.2018.12.022 30634079

[B37] Nawrot J. Napierała M. Kaczerowska-Pietrzak K. Florek E. Gornowicz-Porowska J. Pelant E. (2019b). The anti-serotonin effect of parthenolide derivatives and standardised extract from the leaves of Stizolophus balsamita. Molecules 24, 4131. 10.3390/molecules30071428 31731603 PMC6891796

[B59] Nawrot J. Gornowicz-Porowska J. Nowak G. (2020). Phytotherapy perspectives for Treating Fungal Infections, Migraine, Sebhorreic Dermatitis and Hyperpigmentations with the Plants of the CentaureinaeSubtribe (Asteraceae). Molecules 25, 5329. 10.3390/molecules25225329 33203185 PMC7696306

[B38] Nawrot J. Budzianowski J. Nowak G. Micek I. Budzianowska A. Gornowicz-Porowska J. (2021). Biologically active compounds in Stizolophus balsamita inflorescences: isolation, phytochemical characterization and effects on the skin biophysical parameters. Int. J. Mol. Sci. 22, 4428. 10.3390/ijms22094428 33922647 PMC8122880

[B39] Obeagu E. I. Obeagu G. U. (2024). Breast cancer: a review of risk factors and diagnosis. Med. Baltim. 19 (3), e36905. 10.1097/MD.0000000000036905 38241592 PMC10798762

[B40] Pal M. Das D. Pandey M. (2024). Understanding genetic variations associated with familial breast cancer. World J. Surg. Oncol. 22 (1), 271. 10.1186/s12957-024-03553-9 39390525 PMC11465949

[B41] Pan Z. Zhang H. Dokudovskaya S. (2023). The role of mTORC1 pathway and autophagy in resistance to platinum-based chemotherapeutics. Int. J. Mol. Sci. 24 (13), 10651. 10.3390/ijms241310651 37445831 PMC10341996

[B42] Qiu Y. Yang L. Liu H. Luo X. (2021). Cancer stem cell-targeted therapeutic approaches for overcoming trastuzumab resistance in HER2-positive breast cancer. Stem Cells 39 (9), 1125–1136. 10.1002/stem.3381 33837587

[B43] Ribeiro H. F. Pelloso F. C. Fonseca B. S. D. Camparoto C. W. Carvalho M. D. W. Marques V. D. (2025). Racial and socioeconomic disparity in breast cancer mortality: a systematic review and meta-analysis. Cancers (Basel) 13 (10), 1641. 10.3390/cancers17101641 40427139 PMC12109952

[B44] Rodrigues F. C. Kumar N. V. A. Thakur G. (2019). Developments in the anticancer activity of structurally modified curcumin: an up-to-date review. Eur. J. Med. Chem. 177, 76–104. 10.1016/j.ejmech.2019.04.058 31129455

[B45] Romaniuk-Drapała A. Totoń E. Konieczna N. Machnik M. Barczak W. Kowal D. (2021). hTERT downregulation attenuates resistance to DOX, impairs FAK-Mediated adhesion, and leads to autophagy induction in breast cancer cells. Cells 10 (4), 867. 10.3390/cells10040867 33920284 PMC8068966

[B46] Romaniuk-Drapała A. Totoń E. Lisiak N. Idzik M. Rubiś B. (2025). Telomerase inhibitors TMPyP4 and BIBR 1532 show synergistic antitumor activity in combination with chemotherapeutic drugs. Sci. Rep. 15, 32958. 10.1038/s41598-025-13496-0 41006335 PMC12475129

[B47] Saito K. Arakawa M. Maeda K. Morita E. (2026). Autophagosome marker, LC3, is released extracellularly *via* several distinct pathways. FEBS Open Bio 16 (3), 542–560. 10.1002/2211-5463.70150 41212723 PMC12955746

[B48] Santos S. S. Gonzaga R. V. Scarim C. B. Giarolla J. Primi M. C. Chin C. M. (2022). Lead compound Hy-droxymethylation as a simple approach to enhance pharmacodynamic and pharmacokinetic properties. Front. Chem. 9, 734983. 10.3389/fchem.2021.734983 35237565 PMC8883432

[B49] Sassi K. Nury T. Zarrouk A. Sghaier R. Khalafi-Nezhad A. Vejux A. (2019). Induction of a non-apoptotic mode of cell death associated with autophagic characteristics with steroidal maleic anhydrides and 7β-hydroxycholesterol on glioma cells. J. Steroid Biochem. Mol. Biol. 191, 105371. 10.1016/j.jsbmb.2019.04.020 31034873

[B50] Siswina T. Rustama M. M. Sumiarsa D. Apriyanti E. Dohi H. Kurnia D. (2023). Antifungal constituents of Piper crocatum and their activities as ergosterol biosynthesis inhibitors discovered *via in silico* Study using ADMET and drug-likeness analysis. Molecules 28, 7705. 10.3390/molecules28237705 38067436 PMC10708292

[B51] Tronina T. Bartmańska A. Popłoński J. Rychlicka M. Sordon S. Filip-Psurska B. (2023). Prenylated flavonoids with selective toxicity against human cancers. Int. J. Mol. Sci. 24 (8), 7408. 10.3390/ijms24087408a 37108571 PMC10138577

[B52] Tzenios N. Tazanios M. E. Chahine M. (2024). The impact of BMI on breast cancer - an updated systematic review and meta-analysis. Med. Baltim. 103 (5), e36831. 10.1097/MD.0000000000036831 38306546 PMC10843423

[B53] Uzun K. Bilen C. Y. Yalçın F. N. (2025). Sesquiterpenes and prostate cancer. Phytochem. Rev. 24, 3549–3647. 10.1007/s11101-025-10088-8

[B54] Valentini V. Bucalo A. Conti G. Celli L. Porzio V. Capalbo C. (2024). Gender-Specific genetic predisposition to breast cancer: BRCA genes and beyond. Cancers 16 (3), 579. 10.3390/cancers16030579 38339330 PMC10854694

[B60] Wyganowska-Swiatkowska M. Nohawica M. Grocholewicz K. Nowak G. (2020). Influence of Herbal Medicines on HMGB1 Release, SARS-CoV-2 Viral Attachment, Acute Respiratory Failure, and Sepsis. A Literature Review. Int. J. Mol. Sci. 21, 4639. 10.3390/ijms21134639 32629817 PMC7370028

[B55] Xiong X. Zheng L. W. Ding Y. Yu-Fei C. Yu-Wen C. Lei-Ping W. (2025). Breast cancer: pathogenesis and treatments. Sig. Transduct. Target. Ther. 10, 49. 10.1038/s41392-024-02108-4 39966355 PMC11836418

[B56] Zarei E. Zarei A. Omidkhoda A. (2025). Parthenolide: pioneering new frontiers in hematological malignancies. Front. Pharmacol. 16, 1534686. 10.3389/fphar.2025.1534686 40303928 PMC12037476

[B57] Zhao J. Zhu K. Li N. Xing L. Sheng R. Shen Y. (2025). Synthetic and pharmacological activities of alantolactone and its derivatives. Chem. Biodivers. 22, e202401798. 10.1002/cbdv.202401798 39679983

